# Optimizing spatial survey administration adopting RT-GSCS: A statistical perspective on performance metrics

**DOI:** 10.1016/j.mex.2024.102578

**Published:** 2024-01-23

**Authors:** Yuri Calleo, Francesco Pilla

**Affiliations:** Spatial Dynamics Lab, School of Architecture, Planning and Environmental Policy, University College Dublin, Ireland

**Keywords:** Real-time geo-spatial consensus system, Real-time spatial Delphi, Spatial consensus, Statistical evaluation, Real-Time Geo-Spatial Consensus System (RT-GSCS)

## Abstract

The Real-Time Geo-Spatial Consensus System (RT-GSCS) is a web-based open-source tool designed for the administration of real-time spatial surveys. One distinguishing feature of this system lies in the adoption of the Real-Time Spatial Delphi logic as the main computational algorithm to achieve a convergence of opinions within a group of individuals. The system is available at http://rtgscs.com/ and is particularly instrumental in supporting the decision-making process when spatial data is absent or when user judgments are solicited for territorial actions. Since the release of the beta version, the system has undergone multiple iterations and significant changes, and for this reason, an updated assessment should be conducted. In this paper, we present an in-depth exploration of the latest version of RT-GSCS (v3.0), which includes novel algorithms, statistical analysis, and spatial techniques, optimized for survey administration. The analysis is meticulously conducted by evaluating a comprehensive array of parameters, encompassing performance metrics, spatial elements, security, updates, and privacy assessments. Moreover, a thorough examination of user feedback is presented, enriching the depth of our investigation. This mixed-method approach ensures a robust and updated investigation of the system, facilitating a nuanced comprehension of the process and the attainment of spatial consensus.

• Existing spatial survey administration systems cannot facilitate group collaboration for achieving a spatial consensus on the territory in real-time.
• The RT-GSCS experienced substantial modifications, necessitating a revised technical evaluation from the initial beta version to its current version.
• The proposed method fills the gaps of a technical performance evaluation of the latest version of RT-GSCS, useful for achieving a spatial consensus among a group of individuals with an assessment of the overall performances.

## Graphical abstract

**Figure:**
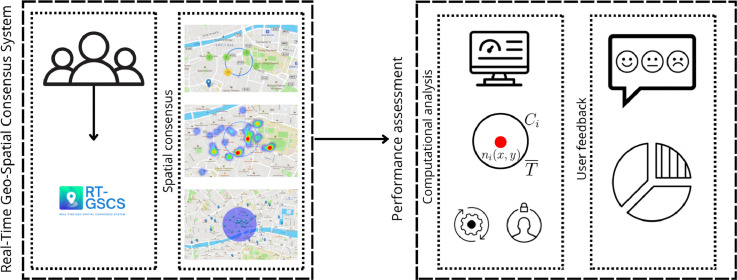

Specifications Table

**Table:** 

Subject area:	Mathematics and Statistics
More specific subject area:	Spatial Statistics
Name of your method:	Real-Time Geo-Spatial Consensus System (RT-GSCS)
Name and reference of original method:	N.A.
Resource availability:	RT-GSCS (https://www.rtgscs.com/)

## Method details

### Background

The “Real-Time Geo-Spatial Consensus System” (RT-GSCS, https://www.rtgscs.com/), represents an innovative web-based open platform developed in 2022 to address the dearth of an efficient technological framework within the scientific literature, capable of obtaining a spatial consensus among a collective of individuals or experts pertaining to a particular subject [1]. RT-GSCS can be defined as a support tool in the decision-making (DM) field, for administering spatial surveys in real-time, adopting different analyses including spatial approaches, statistical indicators, and textual analysis. The system's logic originates from the classical version of the Delphi method, a structured communication technique used to obtain input and opinions from a group of individuals on a particular topic or problem [2]. However, while the traditional Delphi method typically relies on asynchronous communication and multiple rounds of textual surveys [3] – not suitable for spatial contexts – the particularity of the system is the introduction of a unique computational algorithm adapted to spatial contexts, namely the Real-Time Spatial Delphi (RTSD) [4]. Specifically, by adopting the Real-Time Spatial Delphi as the main component of the platform, we give the process dynamism, compared to the standard version of the Delphi method [5], with the final aim to achieve a convergence of opinions among a panel of individuals in a short time frame.

In the current version of the system (v3.0), the contributors can administrate spatial surveys in real-time (also defined as spatial questionnaires), asking different questions to the panel. The panel, generally composed of people with a high degree of expertise on the topic of interest, can at any time, answer the proposed questions anonymously by adding one or more opinion points (N) on an interactive map [6]. From this point, the system calculates an automatic circle showing the degree of convergence on the territory, shrinking, enlarging, and moving in real time based on others’ N judgments. This circle demonstrates exactly the convergence of opinions obtained among the individuals on the territory and can be easily viewed by the panel, both graphically and statistically. Moreover, on the system, different tools are provided to the users in order to give them the opportunity to gather more information, including the possibility of commenting on the opinion-points, specific circle information, spatial analysis (e.g., heat maps, and clustering in real-time) and textual analysis.

In the original method [1], the beta version of the system is illustrated, and the preliminary conditions of the platform are satisfied since it was possible to adopt this novel system to engage a panel of individuals to gather information and administer real-time spatial surveys. Furthermore, from the first version, different case studies have been conducted in different fields including climate change resilience, urban planning, spatial complexity, transportation, society and security, and future studies (e.g., [[1], [7], [8]]).

Specifically, the system demonstrated the utility of a valid tool for decision-making and policy evaluation, especially when quantitative spatial data are either unavailable or only partially available or when expert judgments are required (for further information see [9]). Moreover, after a deep exploration of RT-GSCS by the authors, the tool was demonstrated to be extremely useful for educational contexts, namely in the training of students or citizens to understand more about spatial planning and the use of participatory GIS (PGIS) tools, enabling active cooperation among them. Nevertheless, from the first beta version to the public release, different revisions occurred, specifically optimized to administer spatial surveys, giving the possibility to adopt accurate features, understand more about the process, and enable cooperation among the respondents with advanced tools.

The novelty of this paper lies in a comprehensive assessment of the RT-GSCS and its evolution from the beta version to the current one (v3.0). This assessment includes the development of new computational algorithms and a thorough analysis of the system's overall performance, combining computational metrics and statistical analysis of users’ feedback, thus offering a unique and in-depth perspective on the system's capabilities.

## RT-GSCS functionalities

**Fig. 1:**
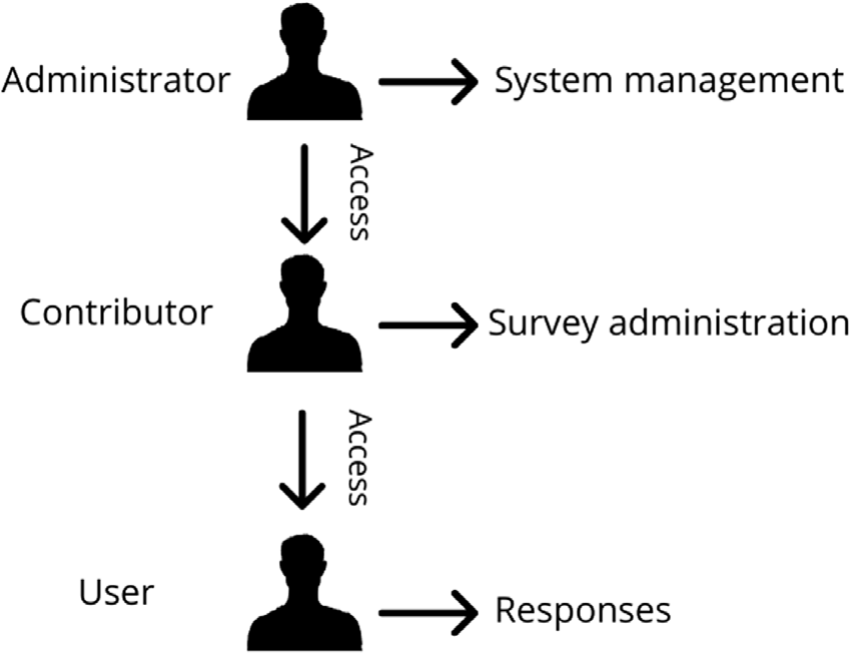
Hierarchical system's organization.

Compared to traditional spatial survey tools available online (e.g., among all ArcGIS Survey123), whose primary purpose is to collect and visualise data from users – performing different specific analyses – but not achieving a consensus among respondents. RT-GSCS pursues a distinct objective, where the main objective of the platform is – as well – to collect and visualize data from users’ judgments, however, with the final aim of obtaining a spatial consensus among them, enabling a dynamic process with active and efficient cooperation. In its latest version (v3.0), RT-GSCS is accessible through web browsers, available at https://www.rtgscs.com/. The RT-GSCS system's organization encompasses three distinct roles with different privileges: 1) *Administrator*: with control panel access and the capability to update the system's source files and codes. 2) *Contributor*: with the capability to craft and administer new surveys after obtaining access from the administrators, incorporate questions, review spatial references, upload attachments, and access statistical summaries. In this case, they do not possess the authorization to alter source files at any time. 3) *User*: with the option to respond to questions put forth by contributors and access all information available within the user interface, including the interactive map, opinion points, circle of convergence, statistical summary, and spatial and textual analysis. In this case, the users do not possess the authorization to access the contributors’ panel, where the surveys’ settings, personal details of the users (e.g., name, surname, emails), or results data are available. In Fig. 1, we depict the RT-GSCS hierarchical system's organization.

**Fig. 2:**
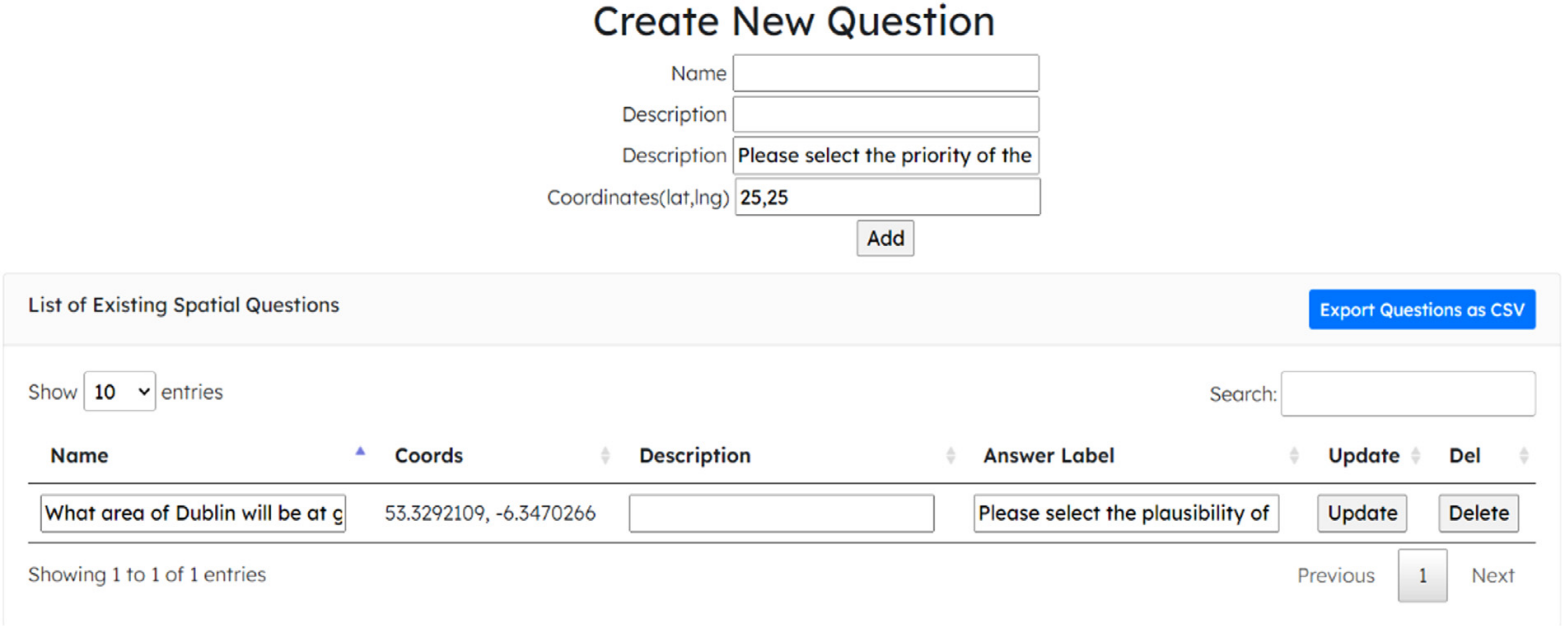
New question submission.

In the original method [1], a customised spatial survey can be drafted when the contributor is granted permission by an administrator. It is possible to upload an introductory section, the relative questions with x,y spatial coordinates, and any supporting materials (e.g., documents, technical guidelines, data, etc.). When the survey is ready to be administered, a personal link is provided automatically and can be shared with the interested panel (from now on, we will refer to the term “panel session” as the transition from a basic survey to a collaborative session). In Fig. 2, we depict the panel where questions can be submitted to users.

**Fig. 3:**
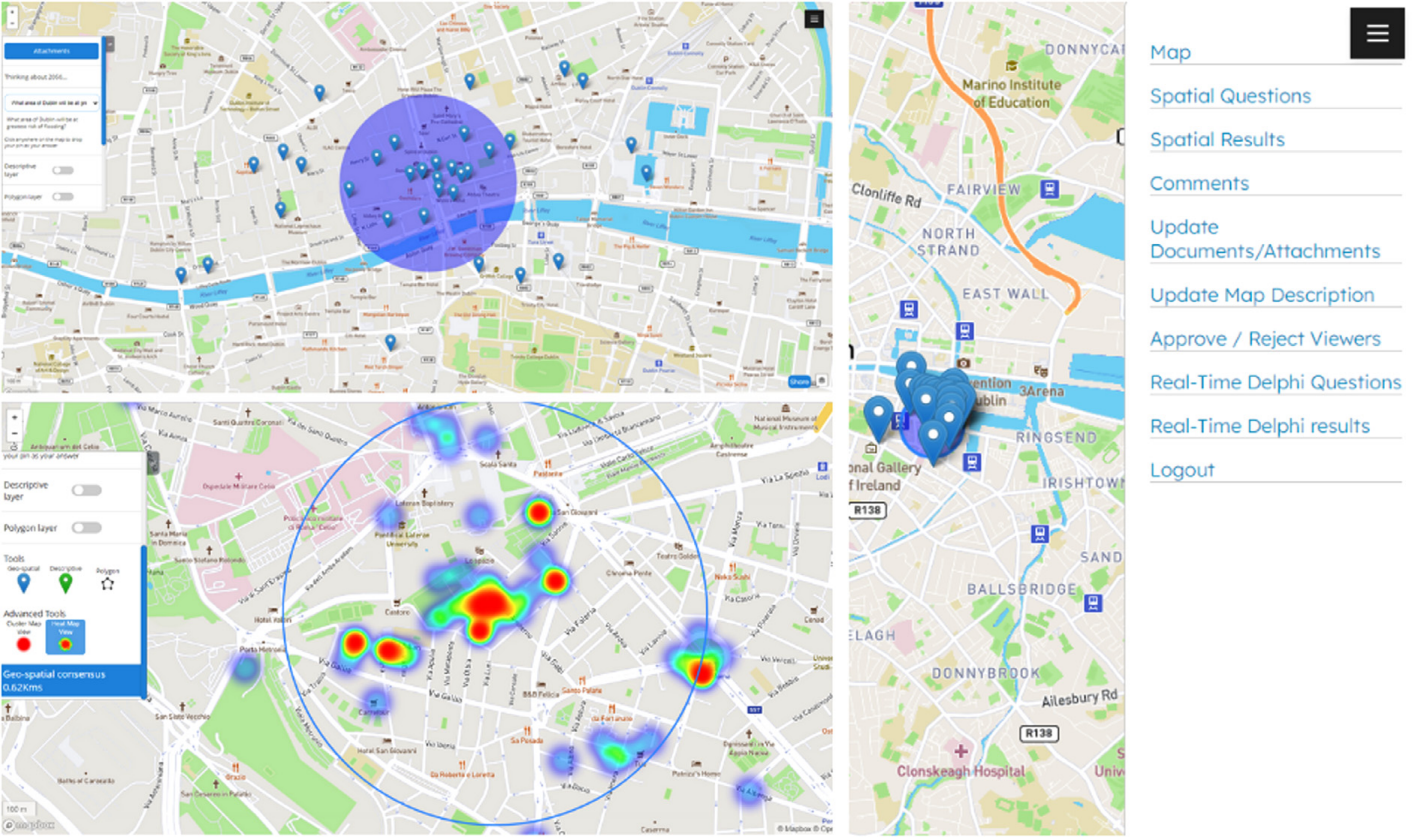
Real-Time Geo-Spatial Consensus System interface.

Users can access the panel session with the private link and register by providing their names and corresponding email addresses. Once approved by the contributors, they can start responding to various questions and actively participate in the survey. As stated in the introductory section, the main interface of the platform is an interactive map, designed to be “user-friendly, for individuals unfamiliar with online spatial survey tools. To answer the different questions, the interface displays a left-hand sidebar, serving as the central hub for all essential information, including attachments, questions, types of opinion points, comments, convergence information, and advanced analysis. As users input their responses in the form of $N_{e}$ points (with e=1,2,…,E the label of the user), the judgments are acquired in the contributors’ panel and are synthesized into the main users’ interface. Users can at any time – and in real-time – place one or more opinion points, add comments, view the statistical summaries, and consult the process of convergence through the consensus circle, thus promoting active participation in the identification of suitable areas of the territory that answer the posed questions. An overview of the main system interface is depicted in Fig. 3.

### Computational algorithms

**Fig. 4:**
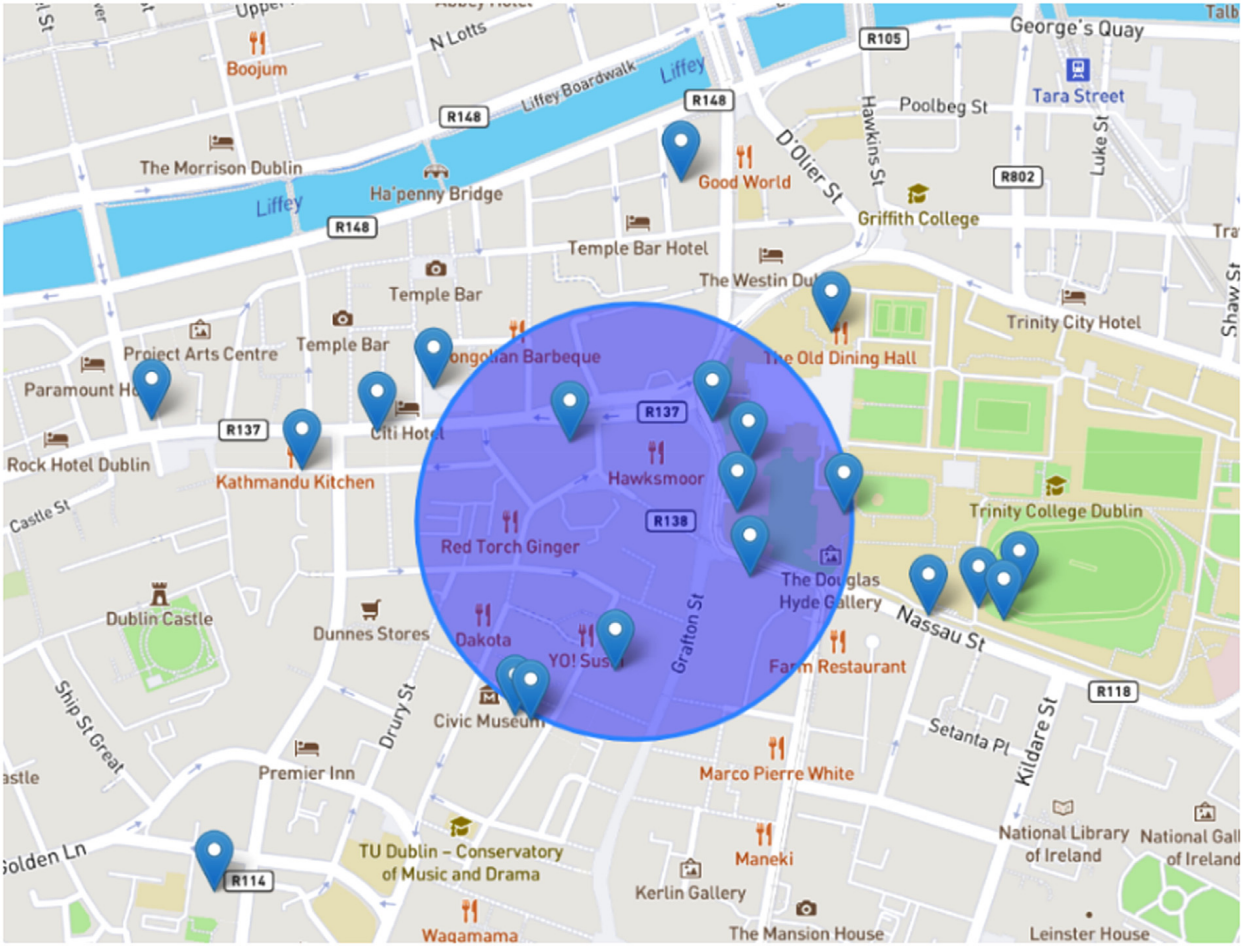
Spatial consensus output.

The algorithm employed in this system is typical of the Spatial Delphi [6] and Real-Time Spatial Delphi [1,4]. Once the users start to answer the different questions on the platform, a set of N judgments are automatically acquired ($N=\sum_{e=1}^{E}N_{e})$. In this case, from the first point placed for each question, the convergence of opinions is highlighted through a geometric element identified as a circle (C). The choice of the geometric element is subjective; however, the circle is a valid element to correctly represent a portion of the territory. In this case, similar to one of the most spread indicators used in the statistical summary of the Delphi method (namely the interquartile range (IQR), the algorithm, find a circle C, the smallest one among the possible $C_{i}$, containing half of the N opinion points. If we follow this logic, a representation of the IQR is pursued in real-time on the interactive map with C, where it includes 50% of the N judgments, with attached the x,y coordinates. During the survey, if the users start to answer to the different questions, we obtain a spatial distribution of $N=n_{1,}n_{2,}\ldots ,n_{N,}$ points on the map. In this case, for each question, the algorithm finds a minimum area $A_{i}$ of the circle $C_{i}$ covering 50% of the points, denoted as:

(1) $$A_{i}⊇T_{N/2}$$

where $T_{N/2}$ is the 50% of the N opinion points. Nonetheless, the primary concern revolves around the existence of an infinite array of possible circle computations, and for this reason, to determine spatial consensus on the map, we consider that $C_{i}$ must have the center in one of the N points. Considering this aspect, the algorithm, calculates for each question a minimum area min(A), denoted as:

(2) min(A)=Med(A)

where for each question, the vector $A=[A_{1},A_{2},...,A_{N}]$, is obtained adopting a matrix M, with all the distances between the N points. The matrix is composed of N columns and N rows, where each intersection value represents the straight-line distance between the points. At this point, the median (Med) of the ordered vector A corresponds to the area $A_{i}$ centred on point $n_{i}$ and containing 50% of the N points. Finally, as specified above, min(A) denotes the spatial consensus obtained on the territory. This logic is adopted in RT-GSCS to enable an active collaboration among the experts or users for immediate decisions. This is because a simple visualization of the N points without a proper convergence circle C, could not lead to a spatial consensus but instead to a static presence of different clusters of points on the territory, thus not allowing for decisions and policy implementations. Considering the traditional version of the Delphi method [10], users can at any time revise their opinions taking into account other users’ feedback, “forcing” the convergence and considering the possibility that some point could be placed outside of the territorial boundaries or in illogic areas. An overview of the spatial consensus output is illustrated in Fig. 4, depicting the N points and the circle C.

The particularity of the v3.0, is the performance of advanced real-time analyses in order to facilitate an immediate view of the N judgments provided by the users, thus enabling an interactive and dynamic process. The analysis implemented include: 1) *Heat map analysis*; 2) *Dynamic cluster analysis*; 3) *Textual analysis*. A heat map analysis is performed in the system to provide insight into the spatial distribution of the N points, with the final objective of identifying hotspots or high-density areas. If we assume that we have a distribution of N points represented by pairs of coordinates (x,y), the algorithm automatically divides the space into a grid of cells, where each cell $G_{\left\{i,j\right\}}$ is associated with a random variable representing the count of data points falling within it ($N_{\left\{i,j\right\}}$). As new data points are collected, these counts are continuously updated in real-time. The heatmap visually reflects this distribution by employing colour intensity to indicate the varying densities of data points across the grid. This advanced analysis is useful to users to have an immediate overview of the opinions on the map, identifying possible hotspots.

The cluster analysis is performed in real-time considering the distance between points and can be defined as a dynamic clustering, as it is interactive and changes in response to user actions, such as zooming in or out. Specifically, let N be the set of data points represented by geographical coordinates on the map, where each $n_{i}$ point has coordinates $n_{i}=\left(x_{i},y_{i}\right)$ for i=1,2,…,N. In this case, if we assume $d_{\left(n_{i},n_{j}\right)}$ as the spatial distance between two data points $n_{i},n_{j}$, we can adopt the following equation to calculate the distance metric adopting Euclidean distance:

(3) $$d_{\left(n_{i},n_{j}\right)}=\sqrt{{\left(n_{i1}-n_{j1}\right)}^{2}+{\left(n_{i2}-n_{j2}\right)}^{2}}$$

**Fig. 5:**
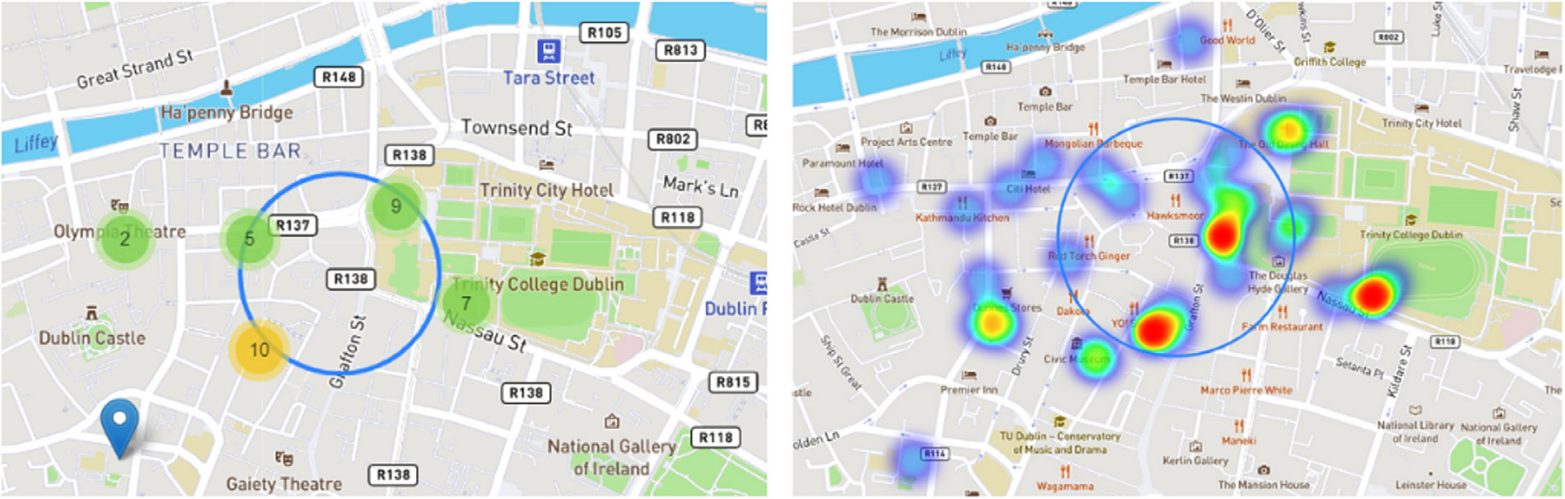
Cluster and heatmap analysis.

The algorithm performs a clustering considering data points based on their spatial proximity. Specifically, we denote S as a set of clusters, where $S_{k}$ is the k-th cluster k=1,2,…,K. Furthermore, since the system incorporates an interactive map, the clustering cannot be static, and change with users’ interaction based on a specific zoom level. Let $K_{\left(z\right)}$ represent the number of clusters when the user is at a specific zoom level z. In this case, $K_{\left(z\right)}$ is dynamic and updates as the user zooms in or out on the interactive map. The assignment of an opinion point $n_{i}$ to a specific cluster $S_{k}$, can be defined as $n_{i}\in S_{k}$, and changes based on the zoom level and the incorporation of the new points on the map (per question). To sum up, we can affirm that Z is the main parameter representing the level of zoom on the map, and can vary continuously, then the dynamic cluster assignment can be expressed as $S_{k_{\left(z\right)}}$, where $n_{i}\in S_{k_{\left(z\right)}}$ means that at zoom level z, data point $n_{i}$ belongs to cluster $S_{k}$. Finally, the visualization of clusters is the visual representation of clusters at zoom level z, involving adjustments in cluster boundaries, colours, and labels. An illustration of the clustering and heatmap analysis is depicted in Fig. 5.

The last analysis performed concerns the users’ comments attached for each question to the N points. In the system, users can at any time comment on other points with the logic of offering collaboration among the parts, thus enabling consideration of the decisions that could be revised by the other users (as in the traditional version of the Delphi method). Frequently, reviewing comments tends to devour a considerable amount of time, especially for survey respondents who have restricted time at their disposal, such as experts, local authorities, or governmental officials. To address this issue, the system employs a textual analysis methodology for each survey question i, adopting the following approach. If we consider a collection of m comments denoted as $Q_{i}$ (with i=1,2,…,m), the algorithm tokenizes each comment into words representing a set of terms $R_{\mathrm{ij}}$ in the i−th comment related to a question. In this case, since some of the words are not significant or may contain not useful text, we adopt a common stop-words list (English language) obtaining a set of $R{{}^{\prime }}_{\mathrm{ij}}$ words. At this point, we calculate the term frequency (TF) for each term in $R{{}^{\prime }}_{\mathrm{ij}}$, obtaining the frequency of term t in comment j related to question i. Finally, the algorithm calculates the total term frequency across all comments related to question i according to the following equation:

(4) $$TF_{\left(t,Q_{i}\right)}=\sum_{j=1}^{n_{i}}(TF\left(t,{R^{\prime }}_{ij}\right)$$

**Fig. 6:**
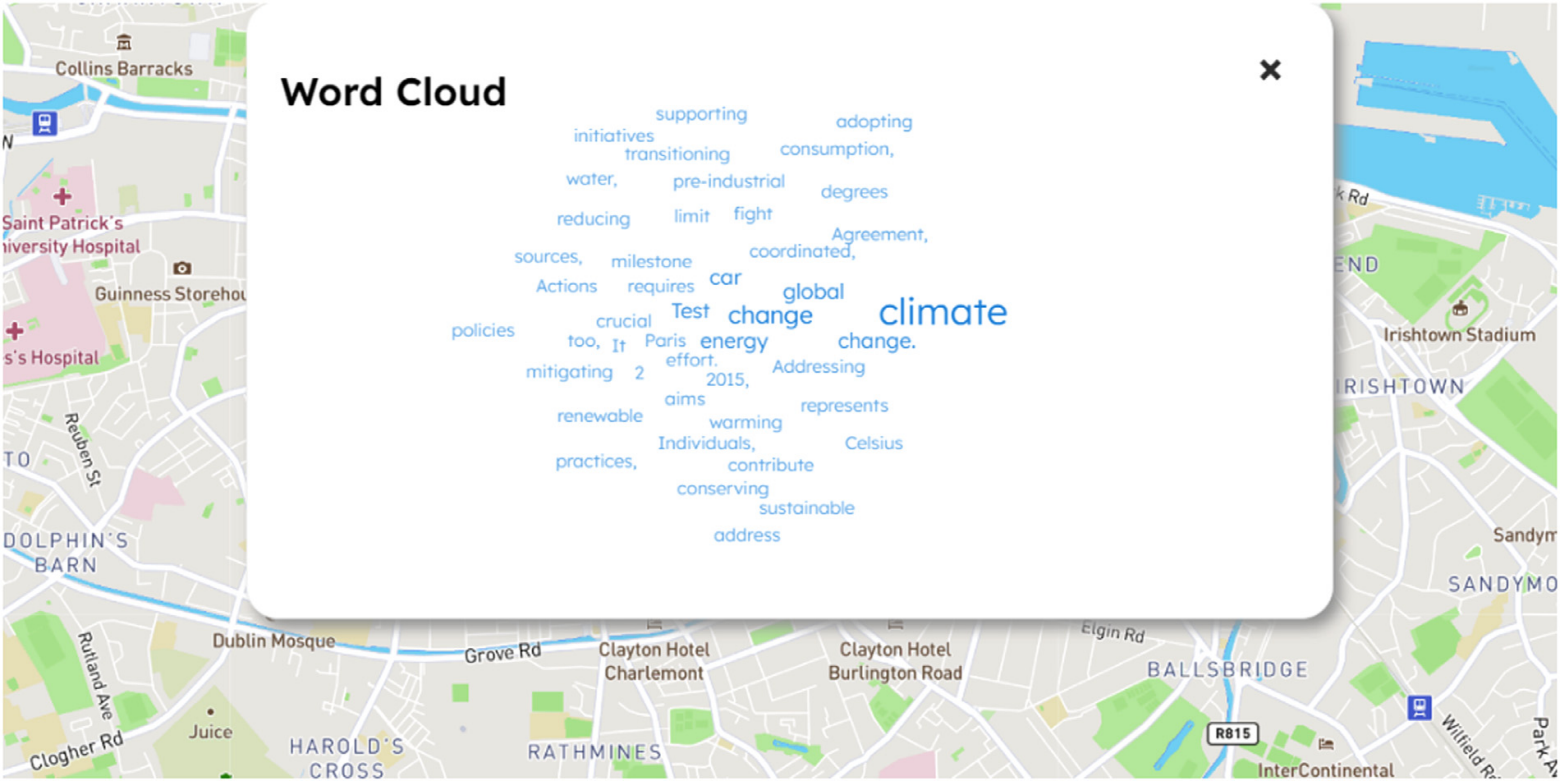
Word cloud analysis.

After the ranking, we consider only the first 50 words in order to depict a real-time word cloud, where the size of each word is proportional to its $TF_{\left(t,Q_{i}\right)}$. With this logic, users can at any time, easily consult an overview of the others’ comments with no efforts. In Fig. 6, the real-time word cloud analysis is illustrated.

## Method and procedure

### Performance assessment

Calleo et al. [1] provide a comprehensive exposition of the beta version, offering a thorough elucidation of the methodology employed for crafting spatial scenarios within the framework of climate change. Nevertheless, from the first version different change occurs and from a technological perspective, it necessitates a dedicated evaluation of its performance, encompassing both computational aspects and a statistical analysis of user feedback. To address this issue, we adopt a mixed-method approach in order to assess the overall system, while considering the opinions gathered from contributors and users. The assessment performed in this paper can be divided into two main tasks: 1) *Performance analysis*: designed to evaluate the system's performance, encompasses aspects like speed, optimization, structural integrity, usage analysis, response time to user input (e.g., the duration between input entry and system display), updates, maintenance, and privacy assessment. From a technical standpoint, it is paramount to guarantee that the system operates in accordance with its design, fully satisfying user expectations and requirements while safeguarding their privacy. 2) *Feedback analysis*: with the ultimate goal of collecting and scrutinizing user feedback, which encompasses ease of use, data integration capabilities, technical support, and consensus convergence. By combining and comparing computational metrics with users’ feedback, we can gain a more comprehensive insight into the system's performance of v3.0.

### System's performance metrics

To assess the performance of RT-GSCS, we adopted GTmetrix (https://gtmetrix.com/) in its recent version. GTmetrix stands as a web performance analysis tool dedicated to evaluating and offering insights into the speed and optimization of websites [11]. It evaluates different aspects, including page loading times and recommends improvements for better web performance. In our specific case, our objective is not to scrutinize the overall performance of the main website. Rather, we aim to assess the system utilized for conducting the spatial survey. Upon logging in, we gain access to the system integrated on the following website: https://rtgscs.com/services/index.php). To have an estimation of the results, we test the system for 15 days, during different active panel sessions, generating a report with an automatic test server adopting as a main browser Google Chrome in different geographic locations. The first analysis performed assesses a combination of the page's speed in terms of loading, interactivity, and visual stability, along with its suitability for optimal performance. In our case, this is useful because it provides an overall – but comprehensive – vision of the system experience, taking into account the front end (really important for those responding to the survey). The main output of the analysis is an overall grade given by the tool defined as “*GTmetrix Grade*” and is determined by a weighted average of two percentage-based scores: the Performance Score (PS) (70%) and the Structure Score (SS) (30%). The PS corresponds exactly to the Lighthouse Performance Score (LPS), introduced by Google and captured during GTmetrix tests adopting a specific browser (in this case Google Chrome), hardware settings, and advanced options (e.g., connection speed, AdBlock, etc.). This score reflects the website's functionality from a user's viewpoint and comprises five indicators, categorized into loading performance (45%), interactivity (30%), and visual stability (25%). The GTmetrix results for our RT-GSCS are reported in Table 4 below.

With regard to the loading performance (45%) the three main metrics adopted are:

• First Contentful Paint (FCP) (10%): a performance indicator gauging the swiftness with which users can access tangible content such as text, images, videos etc. on the website. The fast the page renders the contents, the better is the users’ experience. In this case, it is considered an optimal result when FCP≤934ms.
• Speed Index (SI) (10%): a performance metric adopted in the PS to assess the load speed of the top part of the web page. This is computed through a frame-by-frame analysis of the page loading. We consider an optimal result when SI≤1311ms.
• Largest Contentful Paint (LCP) (25%): defined by Google as a “user-centric” indicators, measures the perceived load speed considering this time the bigger content of the page (e.g., a picture or a carousel) an indicator to evaluate the perception of speed of charge of a web page. In our case, this indicator is particularly important since the bigger content of the page is the interactive map, and we can consider an optimal result when LCP≤1200ms.

With regards the interactivity (30%), GTmetrix adopts the following indicator:

• Total Blocking Time (TBT) (30%): it proves especially valuable for quantifying the overall duration from FCP to the time to interactive, thereby assessing the cumulative period during which your webpage remained inaccessible and hindered user interaction. An optimal result of this indicator is when TBT≤150ms.

Finally, the metric adopted in the PS is the visual stability (25%) with the following indicator:

• Cumulative Layout Shift (CLS) (25%): measures the perceived visual stability of the web page load including “unexpected shifting of web elements in the rendering process” (e.g., loading of specific elements, prioritizing some over others, rather than loading them all simultaneously). In this case, this indicator is a score and is calculated considering the viewport size and the movement of unstable elements in the viewport between two rendered frames. We use the following equation:
  (5) CLS=IF·DF

where IF corresponds to the impact fraction, measuring how unstable elements impact the viewport area between the two frames, and DF is the distance fraction, measuring the displacement of shifting elements in relation to the viewport. We can consider an optimal result when CLS≤0.1.

The above cited metrics are assessed and computed as a numerical score. These scores are subsequently evaluated against predefined thresholds, and when combined with the relevant weightings, they contribute to the ultimate PS.

Within the GTmetrix report and the overall grade, an integral component is the Structure Score [12], which evaluates the webpage's functionality with the aim of achieving optimal performance. This evaluation encompasses personalized GTmetrix optimizations, which involve actions like enabling keep-alive for pages using HTTP/1.1, consolidating images through CSS sprites, and refraining from using CSS @import. These optimizations, in addition to the standard Lighthouse audits by Google Inc., do not impact the Performance Score but serve to highlight structural issues within the webpage. In accordance with the description provided, the weighted average derived from the metrics automatically assigns a letter grade, spanning from A to F, which is then presented on the comprehensive GTmetrix report. In Table 1 we highlight the percentage ranges corresponding to each letter grade.

**Table 1:** GTmetrix percentage ranges.

Grade (%)	Letter Grade
90 – 100	A
80 – 89	B
70 – 79	C
60 – 69	D
50 – 59	E
0 – 49	F

### Spatial convergence elements assessment

Within the dynamic process of administering spatial surveys with RT-GSCS, three key components are crucial for attaining spatial consensus: the spatial coordinates representing user opinions, the circle's variation with respect to point insertion, and user comments on these points. As a result, it follows that, it is imperative to comprehensively assess the system's performance in data acquisition and displaying, since the panel session is completely in real-time and a possible input-output slowness could have implications for the final results. For these reasons, we focus on the assessment of the following parameters:

• Average speed from the insert of a $n_{i}$ point to the effective acquisition and display.
• Average speed from the insert of a comment to the effective acquisition and display.
• Average speed from the effective insert of a $n_{i}$ point to the moment when the circle C is effectively displayed.

To perform this analysis, we rely on the analytics dashboard within our website panel, which provides pertinent information about website usage and metrics. It is crucial to emphasize that administrators do not have direct access to the survey data; access is exclusively granted to contributors, and only for the specific surveys they are involved in. Nonetheless, the system integrates an analytics dashboard that offers a range of aggregated and anonymized data. In our case, this data encompasses the duration between the insertion of a point and its visibility on the map, the elapsed time between comment insertion and its display on the left-hand panel, and the duration from the point's submission to the subsequent adjustment in circle size. To compute the average speed, defined as the time elapsed from insertion to actual submission and display, we employ the following indicator:

(6) $$\bar{T}=\frac{\left(n_{t1}+n_{t1}+\ldots +N_{tN}\right)}{N}$$

where $\bar{T}$ represents the average of the elapsed times, which is obtained by subtracting the times of points acquired from the times of points entered, and then dividing the result by the number of points. Aswell, the same metric is used for comments and for the circle of convergence. In this last case, the sum of the times of points entered and the times of circles displayed is also divided by the number of points. This is due to the assumption that if we consider the circle $C_{i}$ changing after each entry of a point $n_{i}$ (even if just a few $km^{2}$), the sum of all changes in circles is C=N and vice versa.

In the scientific literature, various contributions provide definitions and methodologies for assessing the performance of systems or websites (e.g., [13]). However, our case is specific, and there is no unanimous consensus on the optimal time frame for evaluating the duration between input and output. Indeed, our real-time process involves active participation from experts and users, and it is crucial to validate this aspect to minimize drop-out rates resulting from a sluggish transition from input to output. In any event, we can consider to the metrics that characterize the efficiency of a website in terms of the speed of page loading, window opening, and feature activation [14,15] applied to our case. We propose the following scale to evaluate the input-output speed:

• If $\bar{T}\leq 3sec$, it can be deemed as an optimal outcome.
• If $4sec\leq \bar{T}\leq 6sec$, it can be considered an acceptable result, though it requires enhancement.
• If $\bar{T}\geq 7sec$, it can be considered as an unsatisfactory result.

The rating scale used for assessing input and output speed adheres to the standards expected from a system. In our case, a user's command response is considered optimal if it takes less than 3 seconds, but it starts to raise concerns when it takes 4 seconds or more. It is worth noting, however, that the scale employed does not account for the user's connection quality, as a point inserted at time $t_{i}$ may be acquired with a delay due to issues related to the connection, stability, or latency. Nevertheless, in terms of users’ collaboration, this does not impact the real-time functionality of the system, as other users do not see the point until it is acquired and displayed by the system.

### Updates and privacy assessment

As a component of our performance evaluation, for the assessment of the updates processed from the beta version to the current one, we proceed with an evaluation of the released versions. Updates and maintenance are of paramount importance in the realm of online systems, serving as the foundation for their continued functionality and longevity. However, they necessitate a meticulous assessment to comprehensively understand their evolving contributions and improvements over time. In our scenario, we can readily access a list of the system's versions alongside their corresponding release dates through the analytics panel. An overview of the system's updates with the corresponding released data is provided in Table 2.

**Table 2:** System's updates.

Version	Released date
v1.0	15/02/2022
v1.1	19/02/2022
v1.2	23/02/2022
v1.3	21/03/2022
v1.4	15/05/2022
v1.5	08/06/2022
v1.6	30/10/2022
v2.0	29/01/2023
v2.1	15/03/2023
v2.2	14/03/2023
v2.3	02/05/2023
v2.4	01/06/2023
v3.0	25/06/2023

In particular, we evaluate since 2022, the following elements:

• Updated features: evaluating the tools available within the platform. This aspect can significantly impact the user experience and the quality of spatial surveys. The availability of tools for data analysis, visualization, and manipulation is crucial for spatial survey administration.
• Updated graphical design: including user interface and user experience design. It plays a crucial role in the assessment of the system, where an aesthetically pleasing and user-friendly design can enhance the overall usability of the platform, making it more attractive and intuitive for users. Clear and well-organized spatial survey forms and data visualization tools can improve user engagement and the quality of data collected.
• Updated performance: is a crucial aspect, especially in systems dealing with spatial data. Efficient data processing, rendering of maps, and responsiveness is essential for a seamless user experience. Poor performance can lead to frustration among users, reduced data collection efficiency, and potential data loss.
• Updated security: including the consideration of sensitive or confidential information. Assessing security ensures the protection of user data, prevents data breaches, and maintains user trust.

RT-GSCS was developed to facilitate the administration of spatial surveys, even for individuals without GIS expertise or experience with online platforms. Additionally, since experts and users are often occupied, employing a user-friendly system can significantly enhance the overall process, reducing drop-out rates. For these reasons, the aforementioned elements prioritize a user-centred approach for analysing updates over time, emphasizing the user perspective over a parametric viewpoint.

In this context, another aspect to evaluate in our assessment is the regulation of sensible data acquired from users. Assessing privacy is extremely important in the context of online platforms specifically when data are acquired directly from users, as it directly impacts the trust, integrity, and ethical considerations of the process [16,17]. Ensuring robust privacy measures safeguards individuals’ data, mitigates the risk of data breaches, and maintains compliance with legal and ethical standards. Furthermore, participants are more likely to engage openly and honestly when they trust that their privacy is protected, resulting in more accurate and valuable data. In order to determine the adequacy of privacy conditions and the level of user protection of the current version of RT-GSCS, we suggest an examination of the following parameters:

• Data access controls: to gain insight into the data access phase, particularly with regard to data needed during registration and surveys, this includes the management of the data, the user profiles with access, and the authorized personnel for data viewing.
• Consent mechanisms: to examine the methods employed by the system to obtain informed consent from users for the collection and utilization of their data.
• Data retention policies: to evaluate the duration for which the system retains user data and its compliance with best practices for data retention.
• Data Anonymization: to check if the system anonymizes or pseudonymizes data to protect the identities of users.
• Privacy Policies and Terms of Service: to evaluate the comprehensiveness and clarity of the system's privacy policies and terms of service.
• Compliance with Data Protection Regulations: to ascertain whether the platform adheres to pertinent data protection regulations, such as the General Data Protection Regulation (GDPR).
• Third-Party Data Sharing: to explore whether the platform shares user data with third parties and the circumstances under which such sharing occurs.
• Incident Logging and Monitoring: to verify whether the platform has established mechanisms for logging and monitoring access to user data, enabling the detection and response to unauthorized or suspicious activities.

### Users feedback

Finally, part of our assessment is the evaluation of the users’ feedback over the time. While metrics provide valuable quantitative insights into the performance and effectiveness of RT-GSCS, user feedback offers an equally essential qualitative perspective that enriches the assessment process. Their feedback sheds light on usability issues, user satisfaction, and identifies areas for improvement that metrics alone may not reveal. Combining both metrics and user feedback in the assessment process offers a more holistic view, empowering developers, and administrators to make informed decisions that not only optimize the system's performance but also enhance the overall user experience and user satisfaction.

To meet these objectives, an online survey is administered to contributors and users over the time (from the beta version to the current one), both via email and from the website page (http://www.rtgscs.com/) and is divided into detailed sections: 1) *Respondent details*; 2) *Main interface*; 3) *Functionalities and tools*; 4) *Analysis, visualization, and extraction of data*; 5) *Support team*; 6) *Further improvement*; 7) *Convergence process*. The online survey was submitted, acquiring an overall of N=65 respondents, of which 11 contributors and 54 users. A Likert scale (1-5) is used for closed questions, however open-ended questions are adopted for questions requiring more explanations. In view of the nature of the questions, the results obtained are analysed adopting descriptive statistics to then be compared to the computational methods in order to understand if an agreement can be outlined. Table 3 provides an overview of the questions presented, along with the roles to which they are directed and their respective question types. The table below does not include questions related to personal information or occupational roles.

**Table 3:** Proposed questions.

Number	Proposed questions	Role	Type
1	How user-friendly do you find our platform?	Contributor/User	Scale (1-5)
2	How satisfied are you with the functionality of our platform?	Contributor/User	Scale (1-5)
3	How satisfied are you with the features of our platform?	Contributor/User	Scale (1-5)
4	How easy did you find the visualisation of data?	Contributor/User	Scale (1-5)
5	How easy did you find the extraction of data?	Contributor	Scale (1-5)
6	How likely are you to recommend our platform to a friend or colleague?	Contributor/User	Scale (1-5)
7	How responsive have our customer support team been in addressing your inquiries or issues?	Contributor/User	Scale (1-5)
8	How would you rate the overall value of our platform?	Contributor/User	Scale (1-5)
9	Is there anything you would like to see added or improved on our platform?	Contributor/User	Open
10	Have you experienced any technical issues while using our platform? If yes, please describe.	Contributor/User	Open
11	How would you describe your overall experience using our platform?	Contributor/User	Open
12	Did you achieve a convergence of opinions?	Contributor	Open
13	Based on the method used in your survey, in how many days did you achieve a convergence?	Contributor	Open

## Method validation

### Metrics results

The evaluation of RT-GSCS performance using the GTmetrix tool has yielded remarkably positive outcomes. The system has earned the highest achievable grade, an “A”, underscoring its exceptional optimization for performance. This performance is further exemplified by a perfect score of 100% in the Performance category, which is a testament to the system's outstanding loading speed, interactivity, and visual stability.

Delving into the specifics of the performance metrics, our initial focus centres on the First Contentful Paint. The indicator indicates a duration of FCP=464ms, which notably outperformed the optimal threshold of 934 milliseconds. This swift FCP ensures that users gain rapid access to initial content, including the main map, related questions, attachments and analysis, contributing significantly to an outstanding user experience. The Speed Index indicator emerged to be also performant, with a result of SI=470ms, well below the 1311 millisecond threshold. This, in essence, assures a rapid loading of the webpage's uppermost portion, allowing users to swiftly engage with the primary content of the page (in our case the interactive map). The Total Blocking Time achieved a score of TBT=0ms, signifying an absence of substantial hindrances to user interaction. This metric quantifies the cumulative period during which the system remains inaccessible, thus ensuring a seamless and uninterrupted user interaction experience. Following the performance analysis, the Largest Contentful Paint indicator impressively registers an output of LCP=513ms, once again falling below the optimal threshold of 1200 milliseconds. This indicates the swift loading of the largest page elements, particularly the interactive map in this context, which positively impacts the perceived speed of the system. Finally, the Cumulative Layout Shift boasts a perfect score of CLS=0, emphasizing an exceptionally stable visual experience for users. This metric gauge the perceived visual stability of the system, ensuring the panel session do not unexpectedly shift during the rendering process, consequently fostering a smooth and well user experience.

In addition to these remarkable performance metrics, the system's Structure Score stands at an impressive 94%, underscoring the well-optimized structural aspects of the website, such as HTTP/1.1 keep-alive, CSS sprites, and judicious CSS usage. These structural optimizations collectively augment the system's overall performance and functionality. It is worth noting that the platform's Structure Score, while not affecting the overall grade, does suggest potential optimizations. In this case, it suggests avoiding CSS @import, enabling back/forward cache restoration, ensuring visible text during web font loading, and minimizing unused CSS and JavaScript. In summary, these results collectively signify an exceptionally well-optimized system, offering an outstanding performance that guarantees stable tool for the administration of spatial surveys in real-time. An overview of the results is illustrated in Table 4.

**Table 4:** GTmetrix results.

Parameter	Output (average)	Details
Grade	A	Positive
Performance	100%	Positive
First Contentful Paint (FCP)	464 ms	Positive
Speed Index (SI)	470 ms	Positive
Total Blocking Time (TBT)	0 ms	Positive
Largest Contentful Paint (LCP)	513 ms	Positive
Cumulative Layout Shift (CLS)	0	Positive
Structure	94%	Positive

### Advanced analysis

The advanced analysis performed in this paper to assess RT-GSCS provided an insightful glimpse into the performance of the system's key components. Table 5 demonstrates swift and efficient processing for the essential elements of the system, namely the spatial points, the circle of convergence, the comments, the response time ($\bar{T}$) and the overall positivity or negativity based on the statistical indicators outlined previously. With regards to the spatial points, from the analytics panel we have a total of N=2564 entries, encompassing the overall points placed during the panel sessions. In this case, $\bar{T}$ is denoted as the average time from point insertion to effective display and is remarkably quick, with an average duration of $\bar{T}=2.57$ seconds. This falls comfortably within the optimal range, underscoring the system's proficiency in acquiring and promptly displaying these points on the interactive map. Similarly, for the circle of convergence, we considered C=2564 entries, and the average time from the submission of a new point $n_{i}$ to the effective circle size adjustment C stands at $\bar{T}=0.03$ cent seconds, further highlighting the system's efficiency. Collectively, these results indicate a high degree of effectiveness and speed in processing user inputs and promptly delivering the corresponding outputs, ensuring a seamless and responsive user experience within the system. It is imperative to highlight the paramount significance of this aspect within our system, as the two components (spatial point and convergence circle) operate in tandem throughout the process. Given the real-time nature of our procedure, should either of these components exhibit delays in transitioning from an input x to an output y, it has the potential to exert adverse effects on the outcomes, thereby influencing the convergence process. To provide an example, consider a scenario in which a user answers a question placing a spatial point $n_{i}$ within the pre-existing circle C derived from prior expert assessments (signifying in a concurrence with other users regarding the consensus territorial area). If other users have entered additional points, but the system has not promptly displayed them, the $n_{i}$ point of the first user in question may find itself outside the consensus circle, thereby potentially altering the results significantly when accumulating such types of errors across a substantial number of responses. Hence, it is reassuring that the system boasts such a short response time, thereby mitigating the limitations of the process.

**Table 5:** Speed of use.

Element	Number	Response ($\bar{T}$)	Details
Spatial points	2564	2.57 s	Positive
Circle of convergence	2564	0.03 cs	Positive
Comments	767	0.15 cs	Positive

RT-GSCS was developed for the administration of spatial surveys in complex decision-making contexts, where spatial data is limited, and there is a need for input from participating expert opinions. For this reason, the two preceding components convert expert opinions into spatial points, identifying a common relevance area for potential policy interventions. However, alongside these two prior components, a crucial aspect in the logic of the Delphi method and Real-Time Spatial Delphi, is the input of expert comments. In fact, these comments can reveal opinions and feedback that may not become apparent in the spatial conversion process. For these reasons, users have the opportunity to provide comments on the N points with the aim of offering additional information and, potentially, prompting a review of the opinions of other experts in the field.

In our case, we have a total of 767 comments, and also here the system displays a remarkable efficiency with an average time of just 0.15 cent seconds from insertion to display. This exceptionally fast processing time is well below the optimal threshold of 3 seconds, demonstrating the system's agility in handling comments.

**Fig. 7:**
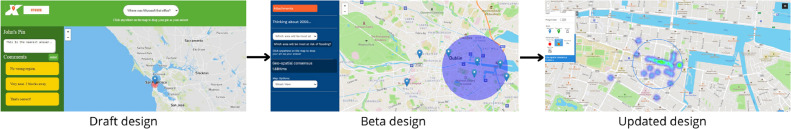
Design updates.

Part of our evaluation is to understand more about the changes over the time from the beta version of the system [1] to the released one. The system development unfolds across several versions, each bringing unique enhancements to cater to user needs. In the system's initial version (v1.0), launched on 15th February 2022, a significant array of enhancements was introduced across multiple facets, encompassing design, features, security, and performance. These pivotal changes ushered in a draft platform design, incorporated a user-friendly map interface, and introduced essential functionalities like questions and point uploading, comment points, and the consensus radius. In this case, the system was a first archetype, and it was in a development process. As the subsequent versions rolled out, starting with v1.1 (19/02/2022), v1.2 (23/02/2022), and v1.3 (21/03/2022), they continued the trajectory of enhancements, albeit in various combinations, consistently focusing on design, security, features, and performance. Whether it was streamlining registration and login processes, fortifying security measures, adding map layers, or enabling real-time features, each iteration underscored the system's unwavering commitment to adapting to the evolving needs of users, all the while ensuring an optimal and secure spatial surveying experience. This commitment to improvement is displayed in subsequent releases. For instance, v1.4, introduced on 15th May 2022, concentrated on design, introducing a new interface design aimed at enhancing both aesthetics and functionality. A graphical overview of the system's interface evolution from v1.0 to v3.0 is presented in Fig. 7, providing a visual depiction to its transformation.

Building upon this foundation, v1.5 (08/06/2022) further addressed design, features, security, and witnessed further enhancements in circle logic, tracking, and overall design elements. V1.6, released on 30th October 2022, continued to emphasize design and security. It introduced login updates and the ability to enable or disable public access, providing users with greater control over system usage. With the advent of v2.0 on 29th January 2023, the system took significant strides in design, features, security, and performance. Users now had the capability to engage in multiple surveys, and registration and login procedures received updates aimed at enhanced security. Version v2.1 (15/03/2023) further expanded on design, features, and security, introducing features like comments tracking, description changes, and password resets to bolster functionality. As the system approached v2.2 (14/03/2023), the focus remained on design and features. Descriptive points and polygons received updates, significantly enhancing data representation and analysis capabilities. Version v2.3 (02/05/2023) introduced innovations such as automatic comment windows when a spatial point it is outside the circle, real-time heat maps and clustering analysis to further elevate data analysis and visualization. With v2.4 (01/06/2023), the system took a leap in textual analysis capabilities, strengthening its capacity for in-depth data understanding. Finally, v3.0 (25/06/2023) integrated real-time textual surveys, providing users with an advanced and comprehensive data collection and analysis experience. Collectively, these versions underscore the system's unwavering commitment to progress, ensuring that users continuously have access to the latest tools, security measures, and performance enhancements to meet their evolving spatial survey needs. The system's evolution represents a dynamic and responsive approach to user feedback, highlighting its position as a cutting-edge solution in the field of spatial surveying. In Table 6. the updates are illustrated highlighting the version number, released date, type of maintenance and further implementation details.

**Table 6:** Updates and maintenance.

Version	Released date	Type	Implementations
v1.0	15/02/2022	Design, Features, Security, Performance	Platform design; Map interface; Questions uploading; Point uploading; Comment points; Consensus Radius;
v1.1	19/02/2022	Design, Security, Performance	Registration and Login Update; Security implementations
v1.2	23/02/2022	Design, Features, Performance	Map layers; Map Attachments; Uploading multiple questions; Delete questions; Modify questions; Backend tracking
v1.3	21/03/2022	Design, Features	Real-Time version; Anonymous comments; Remove all points; Circle logic updates
v1.4	15/05/2022	Design	New interface design
v1.5	08/06/2022	Design, Features, Security	Circle logic update; Circle tracking back-end; Design updates
v1.6	30/10/2022	Design, Security	Log-in update; Enable or disable public access
v2.0	29/01/2023	Design, Features, Security, Performance	Multiple surveys; Registration and log-in updates
v2.1	15/03/2023	Design, Features, Security	Comments tracking; Change description; Remove all points; Reset password
v2.2	14/03/2023	Design, Features	Descriptive points and polygons update
v2.3	02/05/2023	Design, Features	Automatic window for comments outside the circle; Real-Time Heat Map; Clustering
v2.4	01/06/2023	Design, Features	Textual analysis
v3.0	25/06/2023	Design, Features	Integration of real-time textual surveys

### Privacy assessment

At the conclusion of this first section dedicated to a system's evaluation, we propose the results of the privacy assessment. Table 7 includes the main parameters analysed and the data access controls, consent mechanism, data retention policies, data anonymization, the presence of a privacy policy, compliance with DPR, third-party data sharing and incident logging and monitoring. Furthermore, the table illustrates the presence or absence of the parameter in the system.

**Table 7:** Privacy control.

Parameter	Presence
Data access controls	Yes
Consent mechanism	Yes
Data retention policies	Yes
Data anonymization	No
Privacy policy	Yes
Compliance with DPR	Yes
Third-Party Data sharing	No
Incident Logging and Monitoring	Yes

In RT-GSCS, there is a presence of data access controls and a consent mechanism. These controls ensure that data access is restricted to authorized personnel, maintaining the integrity of sensitive user data. The existence of a clear and effective consent mechanism prior to the survey reinforces the platform's dedication to securing informed user consent. In particular – given the hierarchical organizations of roles in RT-GSCS – the data acquired are available only to administrators and developers cannot have access to it. We must emphasize here that the data acquired are not considered sensible since only name, surname and email are collected [18] and managed following GDPR requirements. Furthermore, the data pertaining to each survey administered by the contributors are not displayed to administrators but under the control of the contributors, thus ensuring a strong mechanism of control. In this regard, several data retention policies are activated (e.g., anonymization, emails retention policy, consent records), and the related disclosure that certain user data are retained, adds transparency and accountability. Although data anonymization is not applied for all data, the platform anonymizes personal data, ensuring user privacy in the context of sensitive information. For users, this is particularly important because the process is completely anonymous, and only contributors can access the emails related to the responses. This setup helps avoid any cognitive bias in the real-time process among peers [19]. The availability of a privacy policy on the platform ensures that users are well-informed about data usage, sharing, and protection, contributing to user trust and ethical transparency. At any time, contributors or users may reach out to technical support via email to request the deletion or modification of their personal data. Furthermore, the system's assertion of compliance with data protection regulations, such as GDPR and HIPAA, is commendable as it ensures alignment with global data protection standards (https://rtgscs.com/privacy-policy/). With this regards, the platform's deliberate choice to abstain from participating in third-party data sharing is in full accordance with privacy-centric principles and standards.

Finally, the monitoring of logging is in place, enhancing security by allowing the detection and response to unauthorized or suspicious activities, further fortifying the platform's commitment to data protection and user trust. At the moment, no incident has occurred with the users’ data.

### Users feedback results

**Fig. 8:**
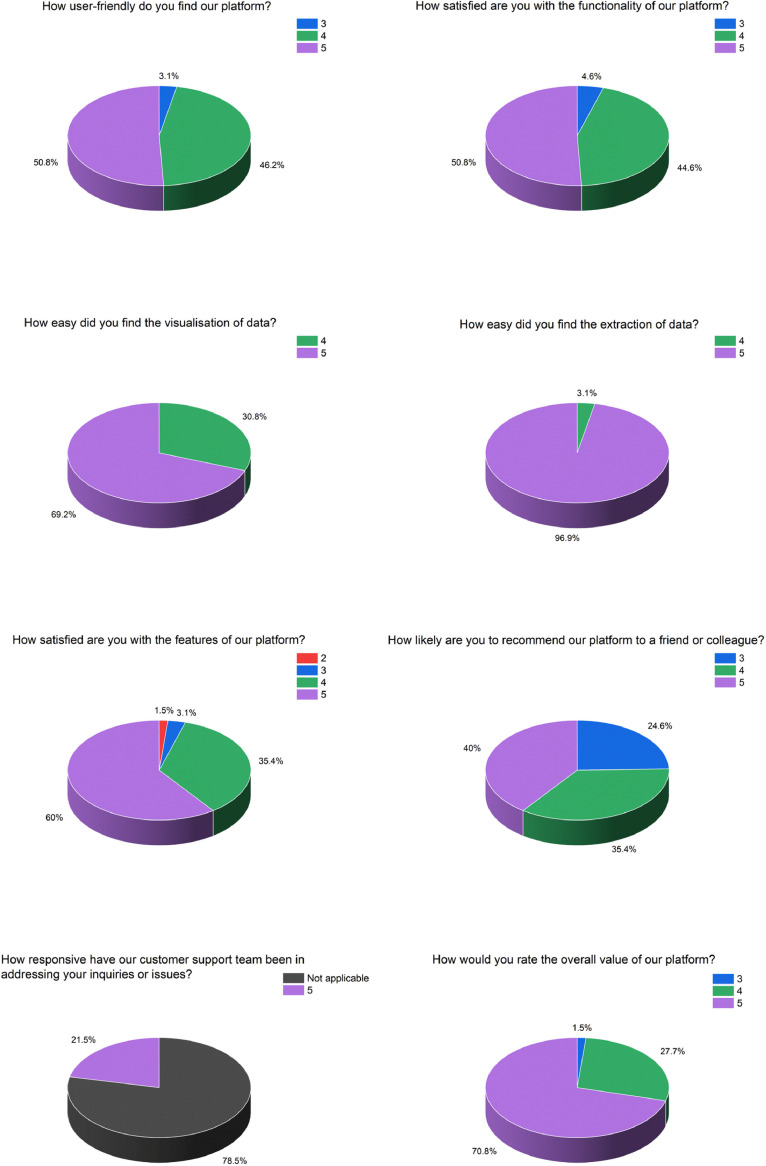
Survey responses.

The other part of our assessment is the analysis of users experience with the survey administration and interaction from the beta version to the current one. The survey is user-centered and encompass different aspects including design, features, visualization and extraction of data and support. In our analysis, we obtained a total of N=65 responses, gaining a better understanding of the participants’ perspectives on various aspects of our platform through a series of questions rated on a scale from 1 to 5 (Fig. 8). The first question, “*How user-friendly do you find our platform?*” garnered significant feedback, with 50.8% of respondents giving the platform the highest possible rating of 5, indicating an extremely user-friendly experience. Furthermore, 46.2% rated it as a 4, signifying a strong degree of user-friendliness, while 3.1% found it to be moderately user-friendly, giving it a score of 3. Moving on to the second question, “How satisfied are you with the functionality of our platform?” similar trends emerged, with 50.8% expressing optimal satisfaction by awarding a rating of 5. Another 44.6% rated it as a 4, reflecting high satisfaction, while 4.6% scored it at 3, indicating moderate satisfaction. The third question delved into data visualization, revealing that 69.2% found the platform's data visualization features outstanding (rated 5), while 30.8% regarded them as highly effective (rated 4). The fourth question focused on the ease of data extraction, and the results were exceptional, with 96.9% of participants awarding a perfect score of 5, highlighting the platform's ease of use for data extraction. Only 3.1% rated it as a 4, indicating a favorable but not ideal experience. The fifth question probed satisfaction with the platform's features. A significant 60% expressed the highest level of satisfaction (rating 5), 35.4% indicated optimal satisfaction (rating 4), 3.1% reported good satisfaction, and 1.5% expressed lower satisfaction. Regarding the sixth question, which asked participants about their likelihood to recommend our platform to others, 40% were highly likely to recommend it, 35.4% were likely to recommend it, and 24.6% fell in the medium recommendation category. Inquiring about the responsiveness of our customer support team, a remarkable 78.5% of participants indicated the highest level of satisfaction, while a 21.5% stated they had not required customer support services. Finally, the last question focused on the overall value of our platform. A majority of 70.8% rated it a perfect 5, signifying high perceived value, 27.7% rated it a 4, indicating a strong value proposition, and 1.5% rated it a 3, implying a moderate level of value. These insightful responses provide a comprehensive understanding of the system's strengths and areas for improvement, helping to tailor the services to better meet the users’ needs and preferences for survey administration.

At this points, open questions are analysed following a discussion approach. The system's users and contributors provided us with valuable feedback, and their responses highlighted both their appreciation for the system's user-friendliness and excellence, as well as their constructive suggestions for improvement. In our analysis, a participant expressed their appreciation for the platform's user-friendliness and suggested the provision of additional tutorial videos or guides specifically tailored for newcomers like themselves, which they believed would be of immense benefit. Another user emphasized their strong desire for the inclusion of more interactive features to augment user engagement and foster a stronger sense of community. Several participants recommended the incorporation of increased customization options to better cater to individual preferences, while others called for a wider variety of content to meet a more diverse range of interests. Furthermore, one user, despite their high overall satisfaction, underscored the potential for a significant improvement by introducing real-time support options for users requiring immediate assistance. In terms of technical issues, the majority of respondents reported smooth experiences with no significant problems. However, a few users did mention minor glitches, occasional delays in loading times, or past login issues. It is important to note that these issues were generally resolved promptly, leading to an overall enhancement in platform performance.

When describing their overall experience, users and contributors consistently used superlatives like “exceptional”, “delightful”, “outstanding” and even “game-changing”. They highlighted the value, enrichment, and enjoyment they derived from the platform, emphasizing the positive impact in decision-making. Regarding the inquiries made to our contributors, their responses indicate a total convergence of opinions, demonstrating the successful realization of the goal within a span of approximately 15–20 days. The feedback and insights provided by the community played a pivotal role in aligning perspectives, nurturing a sense of harmony and unity among our user bases.

Based on user suggestions, over time different implementations have been developed. The system now has targeted tutorial videos and guides designed for newcomers, aiming to facilitate a smoother onboarding experience. Additionally, in line with the desire for increased interactivity and community engagement, we have introduced new features to foster a stronger sense of community within the platform, including the possibility of commenting on others’ judgments anonymously, creating nicknames for each profile, developing a dedicated chat where users can send message each other. Moreover, to address the need for customization options, we have expanded the range of personalization features available to cater to individual preferences, including new designs over time, new tools, specific profile preferences etc. Finally, in response to the request for real-time support options, we have implemented a system to provide quick assistance to users requiring prompt help adopting social network channels and email support. These specific improvements reflect the commitment to actively incorporating user feedback into the ongoing development of the system, ensuring that it evolves to meet the evolving needs and expectations of the user base.

### Conclusion

In this paper, we have presented an in-depth technical examination of the RT-GSCS in its current version (v3.0). We conducted an assessment of its performance metrics, considering both computational aspects and user experience. The system has demonstrated exceptional performance, particularly in decision-making scenarios where spatial data may be absent, effectively transforming user opinions into spatial points. The unique logic underlying the Real-Time Spatial Delphi differentiates its spatial survey administration approach from other existing software/tools solutions, with the primary objective of attaining a spatial consensus. Overall, the metrics’ results demonstrate high performance, with the system achieving favourable scores across all indicators when assessed using the GTmetrix tool. The system appears to be well-optimized in its current version, as evidenced by its swift input-output speed. This optimization enhances the collaborative and cooperative experience during panel sessions. From a security standpoint, it is evident that RT-GSCS has progressively enhanced the platform's features, analysis, design, and other crucial aspects with each new version release. Moreover, the privacy conditions of users are effectively met, with their data being safeguarded through various control mechanisms, all while adhering to GDPR regulations and not being shared with third parties. In conclusion, the feedback provided by users aligns well with the performance metrics, indicating a positive outcome and consistency with the metric results. This reflects the system's strong performance, as endorsed by users who have demonstrated the ability to attain a convergence of opinions in significantly shorter timeframes compared to the traditional version of the Delphi method process.

The constraints highlighted in this paper stem from the evident requirement for thorough validation and empirical support of the system's real-world performance in decision-making scenarios. Nevertheless, this validation has already been demonstrated in Calleo et al. [1]. Additionally, other limitations can be discerned within the general decision-making process, where various cognitive biases may manifest. Nonetheless, this process mitigates some of the cognitive biases due to its anonymous nature. In future endeavours, it is imperative to address these limitations in order to enhance the robustness and efficacy of the RT-GSCS, ensuring a comprehensive evaluation of its capabilities and broader relevance. Continuous feedback mechanisms, internationalization, accessibility, inclusivity, and security enhancements are vital components for the ongoing evolution and success of the RT-GSCS. Future work in these domains will contribute to the system's expansion and its capacity to cater to the diverse requirements of decision-makers, stakeholders, and users across various domains.

## Ethics statements

Not applicable.

## CRediT authorship contribution statement

**Yuri Calleo:** Conceptualization, Methodology, Software, Validation, Formal analysis, Investigation, Visualization, Writing – original draft, Writing – review & editing. **Francesco Pilla:** Conceptualization, Methodology, Supervision, Writing – review & editing.

## Declaration of competing interest

The authors declare that they have no known competing financial interests or personal relationships that could have appeared to influence the work reported in this paper.

## Data Availability

Data will be made available on request.
